# Risk factors of liver metastasis from advanced pancreatic adenocarcinoma: a large multicenter cohort study

**DOI:** 10.1186/s12957-017-1175-7

**Published:** 2017-07-03

**Authors:** Dong S., Wang L., Guo Y. B., Ying H. F., Shen X. H., Meng Z. Q., Chen Hao, Chen Q. W., Li Z. S.

**Affiliations:** 1 Department of Integrative Oncology, Fudan University Shanghai Cancer Center, Shanghai, China; 2 Department of Oncology, Shanghai Medical College, Fudan University, 270 Dong-An Road, 200032 Shanghai, China; 3 Digestive Endoscopy Center, Department of Gastroenterology, Changhai Hospital, The Second Military Medical University, 169 Changhai Road, 200433 Shanghai, China; 4 Department of Integrative Medicine of Shanghai Ruijin Hospital, Shanghai Jiao Tong University School of Medicine, 200025 Shanghai, China; 5 Institute of Clinical Epidemiology, Key Laboratory of Public Health Safety, Ministry of Education, School of Public Health, Fudan University, 200032 Shanghai, China

## Abstract

**Background:**

Clinical prognostic parameters of liver metastasis from pancreatic adenocarcinoma have not been specifically identified.This study is to explore the risk factors of liver metastasis in advanced pancreatic adenocarcinoma (PDAC) patients in China.

**Methods:**

A multicenter cohort study was conducted to explore whether liver metastasis in locally advanced and metastatic PDAC could be reflected by some common laboratory indexes. We collected 1787 advanced PDAC patients from three participating hospitals between 2004 and 2014. The associations between some laboratory indexes and risks of liver metastases were analyzed.

**Results:**

Results have shown that 87% of stage IV patients developed synchronous liver metastasis. Primary tumor location (body/tail vs. head/neck, OR 0.55, 95% CI 0.36-0.83), primary tumor diameter (≥20 mm vs. <20 mm, OR 1.77, 95% CI 1.16–2.70), elevated ALT and AST (OR 1.62, 95% CI 0.92–2.83), and elevated CA19-9 (OR 2.72, 95% CI 1.85–3.99) upon diagnosis are significantly associated with risk of synchronous liver metastasis. Among stage III patients, 30.1% developed metachronous liver metastasis. However, no risk factors were identified among these patients.

**Conclusions:**

Primary tumor location, diameter, elevated ALT and AST, and increased CA19-9 are independent risk factors of synchronous liver metastasis in PDAC patients.

## Background

Pancreatic ductal adenocarcinoma (PDAC) is the fourth leading cause of cancer-related death in the USA [1]. Characterized by the obscure syndromes and delayed manifestation, the prognosis for PDAC is fairly unfavorable. Only 10–20% of the patients can receive radical resection upon diagnosis [2]. Most of patients are diagnosed at advanced stages thus having few chemotherapeutic choices.

PDAC predispose the liver to establish secondary tumors [3]. According to the time when the metastasis is detected, liver metastasis of PDAC can be subdivided into synchronous and metachronous metastasis. Currently, amounts of studies have identified molecules as potential biomarkers predicting liver metastasis in PDAC, which may be of clinical significance [4, 5]. However, most of these biomarkers cannot be detected easily and have not been applied clinically. Currently, clinical prognostic parameters, especially of liver metastasis, have not been specifically identified. Frequently used clinical indexes that can be obtained easily would be of prospective clinical significance in the prediction of liver metastasis.

Herein, we conducted a multicenter cohort study to explore the value of some commonly used laboratory indexes in predicting the liver metastasis in locally advanced and metastatic PDAC patients. Meanwhile, we also report the cumulative metachronous liver metastasis rate for stage III PDAC patients.

## Methods

### Study populations

From January 1, 2004, to December 31, 2014, we conducted a multicenter cohort study in stage III and IV patients with unresectable PDAC at three participating hospitals, Shanghai Cancer Center, Changhai Hospital, and Ruijin Hospital. All the patients were pathologically confirmed with PDAC. In this study, data from 1787 patients in the cohort were collected and analyzed.

Approvals were obtained from the ethics committee at all the three participating centers, Shanghai Cancer Center, Changhai Hospital, and Ruijin Hospital. This cohort study was performed in accordance with the precepts of the Helsinki Declaration. During the hospitalization, written informed consents in view of prospective research of the clinical data were obtained from every included patient or their guardians.

### Exposure measurements

Upon the initial admission to the in-patient department, blood samples were obtained from every patient on empty stomach for 8 h to detect CA19-9, liver function, and serum amylase. Information on smoking status and alcohol consumption was also collected. We defined the normal values of ALB, ALT, AST, and CA19-9 in accordance with the standards stipulated by Fudan University Shanghai Cancer Center. The normal value of ALB ranges from 40 to 55 g/l. The normal ranges of ALT and AST are 9–50 U/L and 15–40 U/L. CA19-9 lower than 27 U/ml was considered to be normal.

### Verification for clinical materials

All the cases were first reviewed by two doctors at each participating hospital, and then, the clinical data was transferred to Shanghai Cancer Center. Two experienced doctors at Shanghai Cancer Center went through these clinical materials again to determine the cases for inclusion. If divergent suggestions were given, a third doctor would be consulted for the final assessment of inclusion or exclusion.

### Outcome ascertainment

#### Synchronous liver metastasis

Up to now, there has been no established definition for synchronous liver metastasis of PDAC. In this study, we defined synchronous liver metastasis as follows:

Synchronous liver metastasis is defined as the detection of liver metastasis upon the initial diagnosis of PDAC. The PDAC should be pathologically confirmed, and the liver metastasis should be validated by imaging examination including ultrasound, CT, MRI, and PET-CT scan.

#### Overall survival and metachronous liver metastasis

All the patients in this study underwent regular follow-up evaluations. Survival status was actively followed up by trained research nurses every month. For deceased patients, dates of death were obtained from their family via telephone call if the patients died after discharge or recorded immediately if the patients died during hospitalization. The follow-up ended after the acquirement of death date. For surviving patients, the data were censored at the last follow-up.

Imaging evaluations were performed every 2 month within the first 3 years and every 3 months thereafter, which include chest X-ray, abdominal ultrasonography, triphasic cross-sectional abdominal CT, and MRI. The images were initially reviewed by an experienced radiologist at each participating hospital and then re-evaluated by a radiologist at Shanghai Cancer Center. If inconsistent evaluations were given by these two doctors, a third radiology specialist at Shanghai Caner Center would be invited to give the conclusive evaluation.

Metachronous liver metastasis is defined as that there is no liver metastasis via imaging examination upon the initial diagnosis of PDAC but was found during the follow-up imaging examinations, regardless of the time after the initial diagnosis of PDAC. For instance, liver metastasis detected 1 month or 12 months after the initial diagnosis of PDAC was both considered as metachronous liver metastasis.

### Statistical analysis

The statistical analyses were performed with SAS statistical software (version 9.4; SAS Institute). A *p* value less than 0.05 (two-sided probability) was interpreted as statistically significant.

Univariate and stepwise multivariate logistic regression models were performed to analyze the association between potential risk factors and synchronous liver metastasis among stage IV patients. Age (≥60 vs. <60), sex (male vs. female), smoking (yes vs. no), alcohol intake (yes vs. no), primary tumor location (body/tail vs. head/neck), primary tumor diameter (≥20 vs. <20 mm), ALT or AST (elevated vs. normal), ALB (low vs. normal), and CA19-9 (elevated vs. normal) were explored as covariables in the models.

Crude metachronous liver metastasis rates of stage III patients were calculated based on the time interval from the diagnosis of pancreatic cancer to liver metastasis ascertained in the follow-up, using the actuarial method commonly performed in colorectal cancers [6]. Patients who died of other diseases were censored at time of death, and patients who developed liver metastasis were censored at the time of occurrence. Univariate and stepwise multivariate Cox proportional hazards regression analyses were performed to analyze the association between potential risk factors and metachronous liver metastasis.

## Results

### Cohort characteristics

This cohort consisted of 652 females (36.5%) and 1135 males (63.5%). Mean age of the patients upon diagnosis was 57.8 years. The numbers of patients at stages III and IV were 707 (39.6%) and 1080 (60.4%), respectively. Detailed baseline characteristics are listed in Table 1.

**Table 1:** Cohort characteristics

	Total	SCC	CHH	RJH
Age, *n* (%)				
<60	946 (52.9)	517 (52.8)	289 (51.2)	140 (57.4)
≥60	841 (47.1)	462 (47.2)	275 (48.8)	104 (42.6)
Sex, *n* (%)				
Male	1135 (63.5)	630 (64.4)	349 (61.9)	156 (63.9)
Female	652 (36.5)	349 (35.6)	215 (38.1)	88 (36.1)
Smoking, *n* (%)				
Yes	849 (47.9)	489 (50.4)	250 (44.6)	110 (45.3)
No	925 (52.1)	482 (49.6)	310 (55.4)	133 (54.7)
Alcohol, *n* (%)				
Yes	242 (13.6)	142 (14.5)	66 (11.8)	34 (14.0)
No	1535 (86.4)	835 (85.5)	492 (88.2)	208 (86.0)
Stage, *n* (%)				
Stage III	707 (39.6)	362 (37.0)	243 (43.1)	102 (41.8)
Stage IV	1080 (60.4)	617 (63.0)	321 (56.9)	142 (58.2)
Primary tumor location, *n* (%)				
Head and neck	762 (42.6)	408 (41.7)	262 (46.5)	92 (37.7)
Body and tail	1025 (57.4)	571 (58.3)	302 (53.5)	152 (62.3)
Primary tumor diameter, *n* (%)				
<20 mm	280 (15.7)	172 (17.6)	76 (13.5)	32 (13.1)
≥20 mm	1507 (84.3)	807 (82.4)	488 (86.5)	212 (86.9)
ALT or AST, *n* (%)				
Normal	1495 (83.7)	813 (83.0)	476 (84.4)	206 (84.4)
Elevated	292 (16.3)	166 (17.0)	88 (15.6)	38 (15.6)
ALB, *n* (%)				
Normal	1377 (77.1)	759 (77.5)	428 (75.9)	190 (77.9)
Low	410 (22.9)	220 (22.5)	136 (24.1)	54 (22.1)
CA19-9, *n* (%)				
Normal	371 (20.8)	214 (21.9)	106 (18.8)	51 (20.9)
Elevated	1416 (79.2)	765 (78.1)	458 (81.2)	193 (79.1)

*SCC* Shanghai Cancer Center, *CHH* ChangHai Hospital, *RJH* RuiJin Hospital

### Synchronous liver metastasis

Among stage IV patients, synchronous liver metastasis status was described at baseline. Synchronous liver metastasis was found in a total of 939 (87%) patients (Table 2).

**Table 2:** Cumulative metachronous liver metastasis rate for stage III pancreatic cancer

	Number	3 months (%)	6 months (%)	12 months (%)
All patients	707	7.2	18.8	37.8
Age				
<60	342	7.7	20.9	41.2
≥60	365	7.0	16.6	35.5
Sex				
Female	293	6.6	20.2	44.5
Male	414	8.0	17.9	33.5
Smoking				
No	381	7.7	19.3	37.1
Yes	323	7.1	18.5	38.1
Alcohol				
No	611	7.5	19.4	37.8
Yes	93	5.5	13.2	40.0
Primary tumor location				
Head and neck	391	7.5	20.5	38.2
Body and tail	316	7.2	16.9	37.5
Primary tumor diameter				
<20	66	4.8	8.6	19.2
≥20	641	7.8	20.1	40.2
ALT or AST				
Normal	605	7.5	18.7	38.4
Elevated	102	6.4	20.0	32.9
ALB				
Normal	563	6.9	18.6	37.9
Low	144	10.2	19.8	37.7
CA19-9				
Normal	132	6.3	15.3	37.6
Elevated	575	7.6	19.7	38.5

Risk factors of the synchronous liver metastasis risk were analyzed in a stepwise multivariate logistic regression model (Table 3). Primary tumor location, primary tumor diameter, elevated ALT or AST, and increased CA19-9 upon diagnosis are significantly associated with synchronous liver metastasis and serve as independent prognostic factors.

**Table 3:** Factors associated with the risk of synchronous liver metastasis for stage IV pancreatic cancer (univariate and multivariate logistic regression model) (*N* = 1080)

Variable	Univariate analysis			Stepwise multivariate analysis		
	OR	95% CI	*P* value	OR	95% CI	*P* value
Age (≥60 vs. <60)	0.94	0.66–1.34	0.7357			
Sex (male vs. female)	1.29	0.90–1.86	0.1723			
Smoking (yes vs. no)	1.19	0.83–1.70	0.3371			
Alcohol (yes vs. no)	1.11	0.65–1.88	0.7058			
Location (body/tail vs. head/neck)	0.59	0.40–0.89	0.0113	0.55	0.36–0.83	0.0046
Diameter (≥20 vs. <20)	1.60	1.07–2.40	0.0236	1.77	1.16–2.70	0.0082
ALT or AST (elevated vs. normal)	1.78	1.03–3.07	0.0390	1.62	0.92–2.83	0.0923
ALB (low vs. normal)	0.80	0.54–1.19	0.2697			
CA19-9 (elevated vs. normal)	2.53	1.74–3.68	<0.0001	2.72	1.85–3.99	<0.0001

### Metachronous liver metastasis

A total of 213 (30.1%) developed metachronous liver metastases among stage III patients during follow up after diagnosis. The overall actuarial cumulative rate was 7.2% after 3 months, 18.8% after 6 months, and 37.8% after 12 months. Table 2 shows the 3-, 6-, and 12-month actuarial cumulative rate according to the characteristics of the patient and of the tumor.

Risk factors of the metachronous liver metastasis were analyzed with a univariate and a stepwise multivariate Cox model to obtain a relative risk of liver metastasis (Table 4). Finally, age and primary tumor diameter were entered into the stepwise multivariate model. However, no statistically significant risk factors were identified in this model.

**Table 4:** Factors associated with the risk of metachronous liver metastasis for stage III pancreatic cancer (univariate and multivariate cox regression model) (*N* = 707)

Variable	Univariate analysis			Stepwise multivariate analysis		
	HR	95% CI	*P* value	HR	95% CI	*P* value
Age (≥60 vs. <60)	0.78	0.60–1.02	0.0736	0.77	0.58–1.01	0.0555
Sex (male vs. female)	0.82	0.63–1.08	0.1534			
Smoking (yes vs. no)	0.98	0.75–1.29	0.8878			
Alcohol (yes vs. no)	0.93	0.63–1.39	0.7288			
Location (body/tail vs. head/neck)	0.81	0.62–1.07	0.1367			
Diameter (≥20 vs. <20)	1.62	1.02–2.59	0.0413	1.58	0.99–2.52	0.0542
ALT or AST (elevated vs. normal)	1.02	0.67–1.55	0.9162			
ALB (low vs. normal)	1.03	0.72–1.47	0.8848			
CA19-9 (elevated vs. normal)	1.15	0.81–1.62	0.4381			

## Discussion

Not surprisingly, hepatic micrometastases are frequently detected in patients with advanced PDAC. By far, few epidemiologic studies have been conducted on the overall metastasis rate of PDAC [7, 8]. Our cohort study has the merit in providing an unbiased and detailed view of the incidence of synchronous and metachronous liver metastases.

Currently, there has been no well-recognized definition on synchronous and metachronous liver metastasis for unresectable PDAC. The definition of synchronous and metachronous liver metastasis varies based on the types of cancers and clinical centers. Synchronous metastases referred to metastases detected before or during surgery for the primary cancer as defined in some studies on colon cancers [9, 10], while defined as metastasis detected within 3, 6, or 12 months after the diagnosis of the primary cancer [11, 12]. The metastases detected after 6 or 12 months following the radical surgery could be viewed as metachronous liver metastasis in gastric cancer [13, 14]. In this study on advanced PDAC, we defined synchronous liver metastasis as the detection of liver metastasis upon the initial diagnosis of PDAC. Metachronous liver metastasis was defined as that there was no liver metastasis via imaging examination upon the initial diagnosis of PDAC, and liver metastases were found during the follow-up imaging examinations, regardless of the time after the initial diagnosis of PDAC. These criteria defined by us differ from those defined in other cancers due to PDAC’s more rapid and aggressive inherent nature as compared with other cancers. However, some small hepatic metastases known as “micrometastases” from PDAC may be overlooked even with advanced imaging and even could be undetected in surgeries [15]. Therefore, the actual number of PDCA patients with liver metastases outnumbers the one identified in this cohort.

Hematogenous metastasis is the most widely accepted theory that cancer cells establish metastases to the liver from advanced PDAC [16]. It has been shown that vascular invasion accounts majorly for liver metastasis [17]. Liver is the first filter for pancreatic cancer cells to invade during the hematogenous metastasis [18]. Pancreatic cancer cells spread to the liver by destroying portal vein, mesenteric vein, and splenic vein thereby achieving hematogenous metastasis. In this study, an intimate association was found between the primary tumor size and the infiltration of pancreatic cancer cells into the liver. There have been quite a few studies demonstrating that the tumor size is the most powerful and reliable predictor of prognosis in resectable patients after radical surgery [19, 20]. This can be easily understood since tumor size directly relates to the T stage and surgical margin [21]. Larger primary tumor size is more invasive to the peripheral organs or vessels and may also mean more tumor burden for the patients [22]. Consequently, we speculated that bigger primary tumor size may bring about more liver metastases among the PDCA patients. In this cohort study, we found that patients with tumor size more than or equal to 2 cm in stage IV patients were more inclined to develop liver metastases, which was not found in stage III patients. This may suggest the minimal influence of the primary tumor size on liver metastases for T4 stage III patients.

Meanwhile, other than the tumor size, primary tumor at the head and neck was more inclined to have liver metastases compared to primary tumor at body and tail for stage IV PDCA patients. However, this result could not be verified in stage III patients, which may be due to the lack of power. Alternatively, the liver metastases in stage III patients are more affected by the inherent aggressiveness rather than the primary tumor size and location.

In this cohort study, patients’ sex, age, smoking status, alcohol consumption, and serum albumin level had no impact on the synchronous metastasis rate in stage IV patients. Neither in stage III patients did these parameters show any influence on metachronous metastasis rate. Mounting evidences demonstrated that some clinical parameters may affect the survival of PDAC patients, such as tumor stages, postoperative pathological findings, and CA-199 level [23–25]. These studies mainly focused on resectable PDAC patients rather than the unresectable ones. While in advanced PDAC, the previous studies mainly concentrated on overall survival [26, 27]. Our study focused on the liver metastases in advanced PDAC and its influencing factors. Primary tumor diameter and elevated CA 19-9 level were found to be independent factors associated with the risk of synchronous liver metastasis for stage IV PDAC. Serum CA 19-9 level is related to the overall tumor burden of PDAC. Patients with elevated CA 19-9 level were found to be in advanced stage [28, 29]. Perioperative CA 19-9 level has been reported to determine the efficacy from radical surgery [30, 31]. A few studies have reported that preoperative CA 19-9 was associated with resectability and postoperative prognosis [32, 33]. This multicenter cohort study demonstrated that patients with elevated CA19-9 level were more inclined to develop liver metastases in stage IV patients. However, CA19-9 level and some other clinical parameters were found not to be risk factors of metachronous liver metastasis for stage III PDAC patients. Therefore, molecular biomarkers other than clinical parameters need to be explored to predict liver metastases in stage III patients.

Serum ALT and AST are commonly used as parameters for liver function [34]. However, they have been rarely reported as prognostic factors for advanced PDAC. Liver functions, indicative of the degree of liver damage, not only reflect liver injury but also reflect cancer cells’ invasion into the liver. To date, studies have shown that elevated expressions of ALT and AST are often associated with poor prognosis in many cancers [35, 36]. Our study suggested that elevated ALT and AST were risk factors for synchronous liver metastasis.

Up to now, surgical resection is the only curative modality for PDAC. Despite improved treatments, the overall survival for PDCA patients is still limited. Due to the aggressiveness of PDAC, multidisciplinary treatment modalities that are of feasibility in advanced colorectal cancer cannot be applied successfully in advanced PDAC. There have been a few attempts clarifying the value of hepatic metastasis resection in advanced PDAC, and prolonged survival has been observed in some cases [37, 38]. However, the numbers of patients enrolled in these studies were too minimal to gain acknowledgement.

Importantly, resection of liver metastases has rarely been clinically performed in advanced PDAC. Currently, there have been mounting clinical trials designed to test the effects of different regimens for advanced PDAC. Unfortunately, most of these attempts resulted in unsatisfactory effects. Recently, two regimens of gemcitabine-nab-paclitaxel and FOLFIRINOX have been demonstrated to improve the overall survival in inoperable patients compared with gemcitabine alone [39, 40]. Results have shown that nab-paclitaxel plus gemcitabine significantly prolonged overall survival for metastatic PDAC patients. Inevitably, peripheral neuropathy and myelosuppression rates were also increased. It also showed that FOLFIRINOX not only improved OS but also entailed increased toxicity [39, 40]. Therefore, these two regimens provide optional treatments for patients with metastatic PDAC but in good performance status.

## Conclusions

From this multicenter cohort study, we found that primary tumor location, diameter, elevated ALT and AST, and increased CA19-9 levels may be independent risk factors of synchronous liver metastasis in PDAC patients.

## Abbreviations

PDAC: Pancreatic adenocarcinoma

## References

[1] Singh D, Upadhyay G, Srivastava RK, Shankar S. Recent advances in pancreatic cancer: biology, treatment, and prevention. Biochim Biophys Acta. 2015;1856:13–27. 10.1016/j.bbcan.2015.04.003 25977074

[2] Stathis A, Moore MJ. Advanced pancreatic carcinoma: current treatment and future challenges. Nat Rev Clin Oncol. 2010;7(3):163–72. 10.1038/nrclinonc.2009.236 20101258

[3] Spadi R, Brusa F, Ponzetti A, Chiappino I, Birocco N, Ciuffreda L, Satolli MA (2016). Current therapeutic strategies for advanced pancreatic cancer: a review for clinicians. World J Clin Oncol.

[4] Yuan Y, Hartland K, Boskovic Z, Wang Y, Walpita D, Lysy PA, Zhong C, Young DW, Kim YK, Tolliday NJ, Sokal EM, Schreiber SL, Wagner BK (2013). A small-molecule inducer of PDX1 expression identified by high-throughput screening. Chem Biol.

[5] Torphy RJ, Tignanelli CJ, Kamande JW, Moffitt RA, Herrera Loeza SG, Soper SA, Yeh JJ (2014). Circulating tumor cells as a biomarker of response to treatment in patient-derived xenograft mouse models of pancreatic adenocarcinoma. PLoS ONE.

[6] Manfredi S, Lepage C, Hatem C (2006). Epidemiology and management of liver metastases from colorectal cancer. Ann Surg.

[7] Wiltberger G, Bucher JN, Krenzien F, Benzing C, Atanasov G, Schmelzle M, Hau HM, Bartels M (2015). Extended resection in pancreatic metastases: feasibility, frequency, and long-term outcome: a retrospective analysis. BMC Surg.

[8] Bouglouga O, Lawson-Ananissoh LM, Bagny A, Kaaga L, Redah D (2015). Pancreatic cancer: epidemiological, clinical, and management aspects in the department of hepatogastroenterology at the Lome Campus teaching hospital (Togo). Med Sante Trop.

[9] Manfredi S, Lepage C, Hatem C, Coatmeur O, Faivre J, Bouvier AM (2006). Epidemiology and management of liver metastases from colorectal cancer. Ann Surg.

[10] Li ZM, Peng YF, Du CZ, Gu J. Colon cancer with unresectable synchronous metastases: the AAAP scoring system for predicting the outcome after primary tumour resection. Colorectal Dis. 2016;18(3):255–63. 10.1111/codi.13123 26400111

[11] Ng WW, Cheung YS, Wong J, Lee KF, Lai PB (2009). A preliminary analysis of combined liver resection with new chemotherapy for synchronous and metachronous colorectal liver metastasis. Asian J Surg.

[12] Wang X, Hershman DL, Abrams JA, Feingold D, Grann VR, Jacobson JS, Neugut AI (2007). Predictors of survival after hepatic resection among patients with colorectal liver metastasis. Br J Cancer.

[13] Hwang SE, Yang DH, Kim CY (2009). Prognostic factors for survival in patients with hepatic recurrence after curative resection of gastric cancer. World J Surg.

[14] Thelen A, Jonas S, Benckert C, Lopez-Hänninen E, Neumann U, Rudolph B, Schumacher G, Neuhaus P (2008). Liver resection for metastatic gastric cancer. Eur J Surg Oncol.

[15] Hatwell C, Zappa M, Wagner M, Michoux N, Paradis V, Vilgrain V, Maggiori L, Panis Y (2014). Detection of liver micrometastases from colorectal origin by perfusion CT in a rat model. Hepatobiliary Pancreat Dis Int.

[16] Kawarada Y, Ishikura H, Kishimoto T, Kato H, Yano T, Kato H, Yoshiki T (2000). The role of sialylated Lewis antigens on hematogenous metastases of human pancreas carcinoma cell lines in vivo. Pathol Res Pract.

[17] Gaujoux S, Allen PJ (2010). Role of staging laparoscopy in peri-pancreatic and hepatobiliary malignancy. World J Gastrointest Surg.

[18] Kamisawa T, Isawa T, Koike M, Tsuruta K, Okamoto A (1995). Hematogenous metastases of pancreatic ductal carcinoma. Pancreas.

[19] Fortner JG, Klimstra DS, Senie RT, Maclean BJ (1996). Tumor size is the primary prognosticator for pancreatic cancer after regional pancreatectomy. Ann Surg.

[20] De Rooij T, Tol JA, Van Eijck CH, Boerma D, Bonsing BA, Bosscha K, Van Dam RM, Dijkgraaf MG, Gerhards MF, Van Goor H, van der Harst E, De Hingh IH, Kazemier G, Klaase JM, Molenaar IQ, Patijn GA, Van Santvoort HC, Scheepers JJ, van der Schelling GP, Sieders E, Busch OR, Besselink MG, Dutch Pancreatic Cancer Group (2016). Outcomes of Distal Pancreatectomy for Pancreatic Ductal Adenocarcinoma in the Netherlands: A Nationwide Retrospective Analysis. Ann Surg Oncol.

[21] Johnston WC, Hoen HM, Cassera MA, Newell PH, Hammill CW, Hansen PD, Wolf RF (2016). Total pancreatectomy for pancreatic ductal adenocarcinoma: review of the National Cancer Data Base. HPB (Oxford).

[22] Wang WL, Ye S, Yan S, Shen Y, Zhang M, Wu J, Zheng SS (2015). Pancreaticoduodenectomy with portal vein/superior mesenteric vein resection for patients with pancreatic cancer with venous invasion. Hepatobiliary Pancreat Dis Int.

[23] Gupta PK, Turaga KK, Miller WJ, Loggie BW, Foster JM (2011). Determinants of outcomes in pancreatic surgery and use of hospital resources. J Surg Oncol.

[24] Liles JS, Katz MH (2014). Pancreaticoduodenectomy with vascular resection for pancreatic head adenocarcinoma. Expert Rev Anticancer Ther.

[25] Wong JC, Lu DS (2008). Staging of pancreatic adenocarcinoma by imaging studies. Clin Gastroenterol Hepatol.

[26] Da Rocha Lino A, Abrahão CM, Brandão RM, Gomes JR, Ferrian AM, Machado MC, Buzaid AC, Maluf FC, Peixoto RD (2015). Role of gemcitabine as second-line therapy after progression on FOLFIRINOX in advanced pancreatic cancer: a retrospective analysis. J Gastrointest Oncol.

[27] Choi Y, Oh DY, Kim K, Chie EK, Kim TY, Lee KH, Han SW, Im SA, Kim TY, Ha SW, Bang YJ. Concurrent chemoradiotherapy versus chemotherapy alone for unresectable locally advanced pancreatic cancer: a retrospective cohort study. Cancer Res Treat. 2015. 10.4143/crt.2015.226 PMC4946358 26511805

[28] Wu L, Huang P, Wang F, Li D, Xie E, Zhang Y, Pan S (2015). Relationship between serum CA19-9 and CEA levels and prognosis of pancreatic cancer. Ann Transl Med.

[29] Matsumoto I, Murakami Y, Shinzeki M, Asari S, Goto T, Tani M, Motoi F, Uemura K, Sho M, Satoi S, Honda G, Yamaue H, Unno M, Akahori T, Kwon AH, Kurata M, Ajiki T, Fukumoto T, Ku Y (2015). Proposed preoperative risk factors for early recurrence in patients with resectable pancreatic ductal adenocarcinoma after surgical resection: a multi-center retrospective study. Pancreatology.

[30] Kanda M, Fujii T, Takami H, Suenaga M, Inokawa Y, Yamada S, Nakayama G, Sugimoto H, Koike M, Nomoto S, Kodera Y (2014). Combination of the serum carbohydrate antigen 19-9 and carcinoembryonic antigen is a simple and accurate predictor of mortality in pancreatic cancer patients. Surg Today.

[31] Nanashima A, Tobinaga S, Abo T, Hatano K, Takeshita H, Nonaka T, Hidaka S, Tanaka K, Kunizaki M, Sawai T, Yasutake T, Nagayasu T (2012). Evaluation of surgical resection for pancreatic carcinoma at a Japanese single cancer institute. Hepatogastroenterology.

[32] Brown EG, Canter RJ, Bold RJ (2015). Preoperative CA 19-9 kinetics as a prognostic variable in radiographically resectable pancreatic adenocarcinoma. J Surg Oncol.

[33] Alexakis N, Gomatos IP, Sbarounis S, Toutouzas K, Katsaragakis S, Zografos G, Konstandoulakis MM (2015). High serum CA 19-9 but not tumor size should select patients for staging laparoscopy in radiological resectable pancreas head and peri-ampullary cancer. Eur J Surg Oncol.

[34] Lai JC, Dodge JL, Sen S, Covinsky K, Feng S (2016). Functional decline in patients with cirrhosis awaiting liver transplantation: results from the functional assessment in liver transplantation (FrAILT) study. [J] Hepatology.

[35] Kim WR, Flamm SL, DiBisceglie AM, Bodenheimer HC, Public Policy Committee of the American Association for the Study of Liver Disease (2008). Serum activity of alanine aminotransferase (ALT) as an indicator of health and disease. Hepatology.

[36] Carr BI, Guerra V (2016). A hepatocellular carcinoma aggressiveness index and its relationship to liver enzyme levels. Oncology.

[37] Ellenrieder V, König A, Seufferlein T. Current Standard and Future Perspectives in First- and Second-Line Treatment of Metastatic Pancreatic Adenocarcinoma. Digestion. 2016;94(1):44–9. 10.1159/000447739 27438590

[38] Lu F, Poruk KE, Weiss MJ (2015). Surgery for oligometastasis of pancreatic cancer. Chin J Cancer Res.

[39] Von Hoff DD, Ervin T, Arena FP, Chiorean EG, Infante J, Moore M, Seay T, Tjulandin SA, Ma WW, Saleh MN, Harris M, Reni M, Dowden S, Laheru D, Bahary N, Ramanathan RK, Tabernero J, Hidalgo M, Goldstein D, Van Cutsem E, Wei X, Iglesias J, Renschler MF (2013). Increased survival in pancreatic cancer with nab-paclitaxel plus gemcitabine. N Engl J Med.

[40] Conroy T, Desseigne F, Ychou M, Bouché O, Guimbaud R, Bécouarn Y, Adenis A, Raoul JL, Gourgou-Bourgade S, De la Fouchardière C, Bennouna J, Bachet JB, Khemissa-Akouz F, Péré-Vergé D, Delbaldo C, Assenat E, Chauffert B, Michel P, Montoto-Grillot C, Ducreux M, Groupe Tumeurs Digestives of Unicancer, PRODIGE Intergroup (2011). FOLFIRINOX versus gemcitabine for metastatic pancreatic cancer. N Engl J Med.

